# Unmasking Tinea Incognito: Case Study, Insights Into the Pathogenesis, and Recommendations

**DOI:** 10.7759/cureus.72042

**Published:** 2024-10-21

**Authors:** Diana Gallegos Espadas, Jesús Iván Martínez-Ortega, Dianely Anahi Garcia Hernandez, Cynthia P Sánchez Mendieta, Ilse Fernández-Reyna

**Affiliations:** 1 Internal Medicine, Hospital Regional Elvia Carrillo Puerto, Instituto de Seguridad y Servicios Sociales de los Trabajadores del Estado (ISSSTE), Mérida, MEX; 2 Internal Medicine, Facultad de Medicina de la Universidad Autónoma de Yucatán, Mérida, MEX; 3 Dermatology, Dermatology Institute of Jalisco, Zapopan, MEX; 4 Histology, Autonomous University of Nuevo León, Monterrey, MEX; 5 Internal Medicine, "Dr. Antonio González Guevara" Civil Hospital, Nayarit, MEX; 6 Mycology, Dermatology Center of Yucatán, Mérida, MEX

**Keywords:** antifungal immune response, atypical tinea, dermatophyte infection, dermatophytosis, insulin resistance, obesity, pathogenesis, steroid-modified tinea, superficial skin fungal infection, tinea incognito

## Abstract

Tinea incognito (TI) is a dermatophyte infection that often presents atypically due to the inappropriate use of corticosteroids or other immunosuppressive treatments, complicating its diagnosis and management. This case report describes a 29-year-old American Indian (Maya) female from Yucatán, Mexico, initially diagnosed with inverse psoriasis and treated with topical corticosteroids. Over several months, her condition deteriorated, with lesions spreading and worsening, ultimately revealing TI. The misdiagnosis was attributed to the masking effects of corticosteroids, which suppressed the immune response and facilitated fungal dissemination.

The case underscores the diagnostic difficulties of TI, particularly when treatments exacerbate rather than alleviate the condition. Key to diagnosis is the combination of patient history, mycological testing, and clinical examination. The study also highlights the role of chronic glucocorticoid use in impairing antifungal immunity by reducing crucial cytokines like IL-17 and IFN-γ, leading to persistent fungal infections. Furthermore, addressing underlying conditions such as obesity, insulin resistance, and diabetes is essential for effective management. Timely and accurate identification of TI, coupled with appropriate treatment, is critical to prevent complications and improve patient outcomes.

## Introduction

Dermatophytes are specialized fungi with a preference for keratin, and they are key agents in causing skin infections, commonly referred to as tineas [1]. Recently, they have been reclassified into nine genera, with *Trichophyton*, *Epidermophyton*, *Nannizzia*, and *Microsporum *being the most common [2].

Tinea incognito (TI) is a dermatophyte infection that has been altered by the inappropriate use of corticosteroids or other immunosuppressive treatments, leading to atypical presentations that often complicate diagnosis [3]. It was initially described in 1968 by dermatologists Ive and Marks [4]. This condition is frequently misdiagnosed as other dermatological disorders such as psoriasis, eczema, or seborrheic dermatitis due to its lack of classic features like central clearing and prominent scaling. The misuse of corticosteroid-based topical medications is a significant contributing factor, as these treatments suppress the immune response, allowing the fungal infection to disseminate while masking its clinical signs [3].

In this case report, we present a 29-year-old Amerindian (Maya) female from Yucatán, Mexico, whose chronic pruritic skin lesions were misdiagnosed as inverse psoriasis and treated with topical corticosteroids. Over several months, the patient’s condition worsened, with lesions spreading to various parts of the body, a hallmark sign of TI.

This case underscores the importance of timely diagnosis and highlights the pathophysiological mechanisms involved. Corticosteroids not only mask clinical features but also facilitate fungal spread by weakening the local immune response. Recognizing TI early enables proper antifungal treatment, preventing further complications. Identifying underlying conditions, such as insulin resistance, is also crucial as they may exacerbate the infection and contribute to its persistence.

## Case presentation

A 29-year-old American Indian (Maya) female from Yucatán, Mexico, with no significant medical history, presented with pruritic skin lesions that had developed over six years. Initially, the lesions appeared in the axillary, inguinal, and inframammary regions. Diagnosed with inverse psoriasis by a general practitioner, she was prescribed a topical corticosteroid. Over several months, she used the treatment intermittently, gradually increasing the frequency due to decreasing effectiveness. Despite this adjustment, the lesions spread, and the itching persisted.

**Figure 1:**
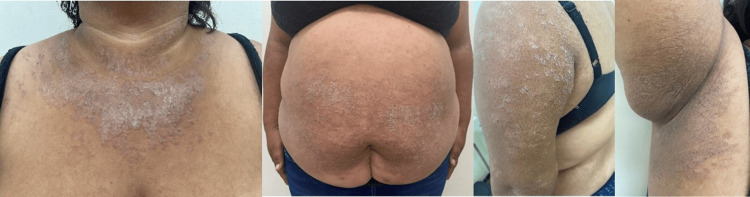
Clinical examination Erythematous-squamous plaques with mild scaling, located on the neck, abdomen, axillae, groin, and posterior side of the arms.

On physical examination, the patient had a body mass index (BMI) of 42, which classifies her as severely obese. Disseminated hyperpigmented erythematous-scaly plaques were observed on the trunk, upper extremities (notably in the axillae), abdomen, and groin (Figure 1).

**Figure 2:**
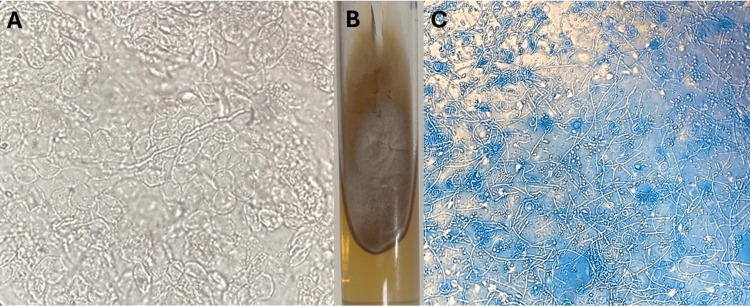
Mycological Examination (A) Direct microscopy of a lesion with 10% KOH (40x): Hyaline hyphae consistent with dermatophytes are observed. (B) Mycological culture on Sabouraud agar: A white, flat, powdery colony typical of *Trichophyton mentagrophytes.* (C) Direct microscopy of the culture with azul de algodon (20x): Multiple microaleurioconidia are observed, further supporting the identification of *Trichophyton mentagrophytes.*

A direct examination of skin scales using potassium hydroxide revealed abundant branching hyaline hyphae consistent with dermatophytes (Figure 2A). The diagnosis of disseminated tinea corporis was confirmed, and treatment with oral itraconazole 100 mg daily for four weeks was initiated.

Laboratory studies, including serological tests for HIV, hepatotropic viruses, and syphilis, as well as autoimmune markers, were all negative or unremarkable. The HOMA-IR (Homeostasis Model Assessment of Insulin Resistance) index was 4.5, suggesting insulin resistance (Table 1).

**Table 1:** Laboratory studies with reference ranges for comparison. This table presents the laboratory findings for the patient, including serological tests for HIV, hepatotropic viruses, syphilis, autoimmune markers, and relevant metabolic indicators. The HOMA-IR (Homeostasis Model Assessment of Insulin Resistance) index was elevated, suggesting insulin resistance, while all other results were within normal reference ranges or unremarkable.

Test	Result	Reference range	Comments
HIV serology (ELISA)	Negative	Negative	No evidence of HIV infection
Hepatitis B surface antigen (HBsAg)	Negative	Negative	No evidence of hepatitis B
Hepatitis C antibody (Anti-HCV)	Negative	Negative	No evidence of hepatitis C
Syphilis serology (RPR/VDRL)	Negative	Negative	No evidence of syphilis
Antinuclear antibodies (ANA)	Negative	Negative	No autoimmune disorder detected
HOMA-IR index	4.5	< 2.9	Indicates insulin resistance
Fasting blood glucose	95 mg/dL	70-100 mg/dL	Within normal range
Hemoglobin A1c (HbA1c)	5.6%	4.0-5.6%	Upper limit of normal, no diabetes
C-reactive protein (CRP)	< 1.0 mg/L	< 5.0 mg/L	No significant inflammation
Complete blood count (CBC)	Normal	WBC: 4.0-11.0 x10^9/L	No evidence of infection or anemia
		RBC: 4.5-6.0 x10^12/L	
		Hemoglobin: 12-16 g/dL	
Liver function tests (LFTs)	Normal	ALT: 7-56 U/L	No liver dysfunction detected
		AST: 10-40 U/L	
Kidney function (serum creatinine)	0.9 mg/dL	0.6-1.2 mg/dL	Normal kidney function

A mycological culture identified *Trichophyton mentagrophytes* (Figures 2B, 2C). Following the completion of the treatment with oral itraconazole, the patient achieved remission of the skin lesions without complications.

## Discussion

Reports on the prevalence of TI vary widely. Some studies suggest that TI accounts for as much as 40% of dermatophytoses, while others report it as very rare, comprising less than 1% of global dermatophyte cases [5]. This discrepancy may be attributed to underreporting and frequent misdiagnosis. A key issue is the incorrect categorization of cases: TI is often mislabeled as “atypical tinea.” While both TI and atypical tinea deviate from the typical ringworm clinical presentation, they differ in their underlying causes. The term “tinea incognito” specifically refers to dermatophyte infections altered by the use of immunosuppressive treatments, particularly oral or topical corticosteroids, whereas “atypical tinea” refers to fungal infections in immunocompetent individuals without the influence of such drugs. TI, therefore, arises as a direct side effect of inappropriate or excessive use of topical or systemic corticosteroids, which mask the characteristic symptoms of the infection [1,5]. We conducted a literature search in PubMed and Google Scholar for prospective or retrospective studies published in the last 10 years to provide a visual and updated overview of clinic-epidemiological data on TI (Table 2) [5-8].

**Table 2:** Summary of clinical and epidemiological studies on tinea incognito (TI) from the last decade

Author	Type of study	Study location	Clinical presentation	Topography	Type of immunosuppressant	Organisms involved	Duration of steroid use	Associated comorbidities
Hyun-Bin Kwak et al. (2023) [8]	Retrospective study	Korea	Eczema 47.4%, rosacea 15.8%, psoriasis 10.5%, systemic lupus erythematosus 10.5%, cellulitis 7.9%, folliculitis 7.9%	Localized (facial)	Topical steroid/calcineurin inhibitor: 55.3%, systemic steroid: 10.5%, systemic steroid and topical/topical calcineurin inhibitor: 18.4%, systemic cyclosporine/topical methotrexate and steroid: 7.9%	Trichophyton rubrum: 26.3%, Microsporum canis: 18.4%, Trichophyton mentagrophytes: 5.2%, Trichophyton verrucosum: 5.2%	3.4 months	Hypertension 21.0%, diabetes mellitus 15.7%, thyroid disease 10.5%, hepatitis 10.5%, angina pectoris 7.9%, dyslipidemia 7.9%, hematological abnormalities 7.9%, asthma 5.2%, neoplasia 5.2%, rheumatoid arthritis 2.6%, others 33.3%
Sharquie et al. (2021) [7]	Prospective study	Iraq	Psoriasis 50%, dermatitis 21.4%, photosensitivity 4.8%, keratoderma 3.4%, seborrheic dermatitis 2.8%, others	Body tinea 35%, facial tinea 24.8%, hand tinea 15%, head tinea 11.8%	Unspecified topical steroids	Trichophyton mentagrophytes	2 weeks to 6 months	Unknown
Dhaher et al. (2020) [5]	Prospective study	Iraq	Eczema 67%, intertrigo 18%, psoriasis 15%, others	Trunk and extremities tinea 33%, groin 24%, hands 22%, facial 13%, scalp 4%	Topical steroid 54.4%, mixed medications 36.6%, calcineurin inhibitors 5.5%, calcipotriol/betamethasone 3.3%.	Unknown	Unknown	Unknown
Dutta et al. (2017) [6]	Prospective study	India	Eczema in most cases followed by inflammatory and autoimmune conditions	Facial tinea 51 cases, tinea corporis 24 cases, tinea cruris 22 cases, facial tinea/tinea corporis 24 cases, tinea corporis/tinea cruris 22 cases, facial tinea/tinea corporis/tinea cruris 13 cases	Triple medication mix in most cases, followed by a combination of betamethasone with clobetasol.	Trichophyton rubrum 35 cases, Trichophyton mentagrophytes 22 cases, Trichophyton tonsurans 3 cases	1.5 months to 12 months	Unknown

In a study on cutaneous adverse drug reactions, TI was identified as the most frequent condition, occurring in 36% of cases. The predominant reason for the misuse of topical corticosteroids was undiagnosed tinea, accounting for 76.7% of cases [9,10]. This suggests that patients or healthcare providers may have used steroid-containing creams under the assumption that the antifungal component would effectively treat the tinea. Consequently, the presence of steroids may have masked the typical signs of the fungal infection, leading to the development of TI. While this hypothesis aligns with the observed data, further research is necessary to confirm this interpretation and to understand better the underlying mechanisms behind the misuse of these treatments.

In addition to the well-established risk factors of immunosuppression, diabetes mellitus, and long-term corticosteroid use, there is still a gap in understanding other contributors to dermatophyte infections. A recent study, which analyzed data from 4,532,665 individuals, revealed that after adjusting for confounding factors, conditions such as dyslipidemia, hypertension, increased alcohol consumption, frequent exercise, a history of smoking, and obesity measured by BMI and waist circumference also significantly raised the risk of developing dermatophyte infections. The authors noted that while exercise emerged as a risk factor, this is likely due to the moist environment created by sweating during physical activity. However, since obesity is a key contributor to dermatophyte infections and exercise is essential for weight management, physical activity should not be discouraged. Instead, the focus should be on proper hygiene practices, such as staying clean and dry after exercise, to reduce the risk of infection [11].

TI typically presents with less pronounced plaques, and minimal scaling, and often lacks the characteristic central clearing associated with the “ring-like” appearance [3,5]. These features make differential diagnosis challenging, leading Samer Dhaher to refer to it as the “new imitator” [5]. A recent systematic review involving 867 patients found that eczema-like lesions were present in nearly 50% of cases, followed by psoriasis (6.57%), pyoderma (4.95%), rosacea (4.95%), and seborrheic dermatitis (4.15%). The review covered 37 differential diagnoses, with the most common locations being the trunk, extremities, and areas with folds, such as the groin [5].

In this case, the lesions initially appeared in intertriginous areas. The differential diagnosis for such conditions includes erythrasma, candidiasis, contact dermatitis, inverse psoriasis, and others [12]. However, we want to emphasize another important consideration for intertriginous lesions, especially when more than two areas are affected: Symmetrical Drug-Related Intertriginous and Flexural Exanthema (SDRIFE). This drug reaction necessitates the immediate discontinuation of the offending medication. Although SDRIFE is typically easier to differentiate due to its acute onset, as opposed to the chronic nature of tinea incognito, prompt recognition remains critical. Notably, cases of SDRIFE following corticosteroid and azole use have been reported [13,14].

Although the typical “ring-like” lesions may be absent, the patient's history remains crucial for diagnosis. Specifically, a history of glucocorticoid use initially showing slight improvement followed by a sudden worsening and spread of skin lesions should raise suspicion. In such cases, additional diagnostic tools like dermoscopy, UV light examination, and mycological studies should be used if available [3].

The extent to which dermoscopic features are altered by steroid use is still unknown. However, it is recommended to perform a dermoscopy and look for typical signs in both glabrous and hairy skin. In glabrous skin, these include dotted vessels, superficial white scales with a peripheral distribution, and a “moth-eaten” appearance with outward-peeling scales. In hairy skin, key features to observe are comma hairs, corkscrew hairs, zigzag hairs, Morse code hairs, black dots, and broken hairs [3,15].

Mycological studies, particularly the potassium hydroxide (KOH) test, are fast, reliable, easy to perform, and cost-effective. In a study of 187 samples tested with 10% KOH, 171 (91%) were positive for hyphae, either mycelium or hyphal forms. Mycological culture is a useful practice that confirms the diagnosis and identifies the species [16].

**Figure 3:**
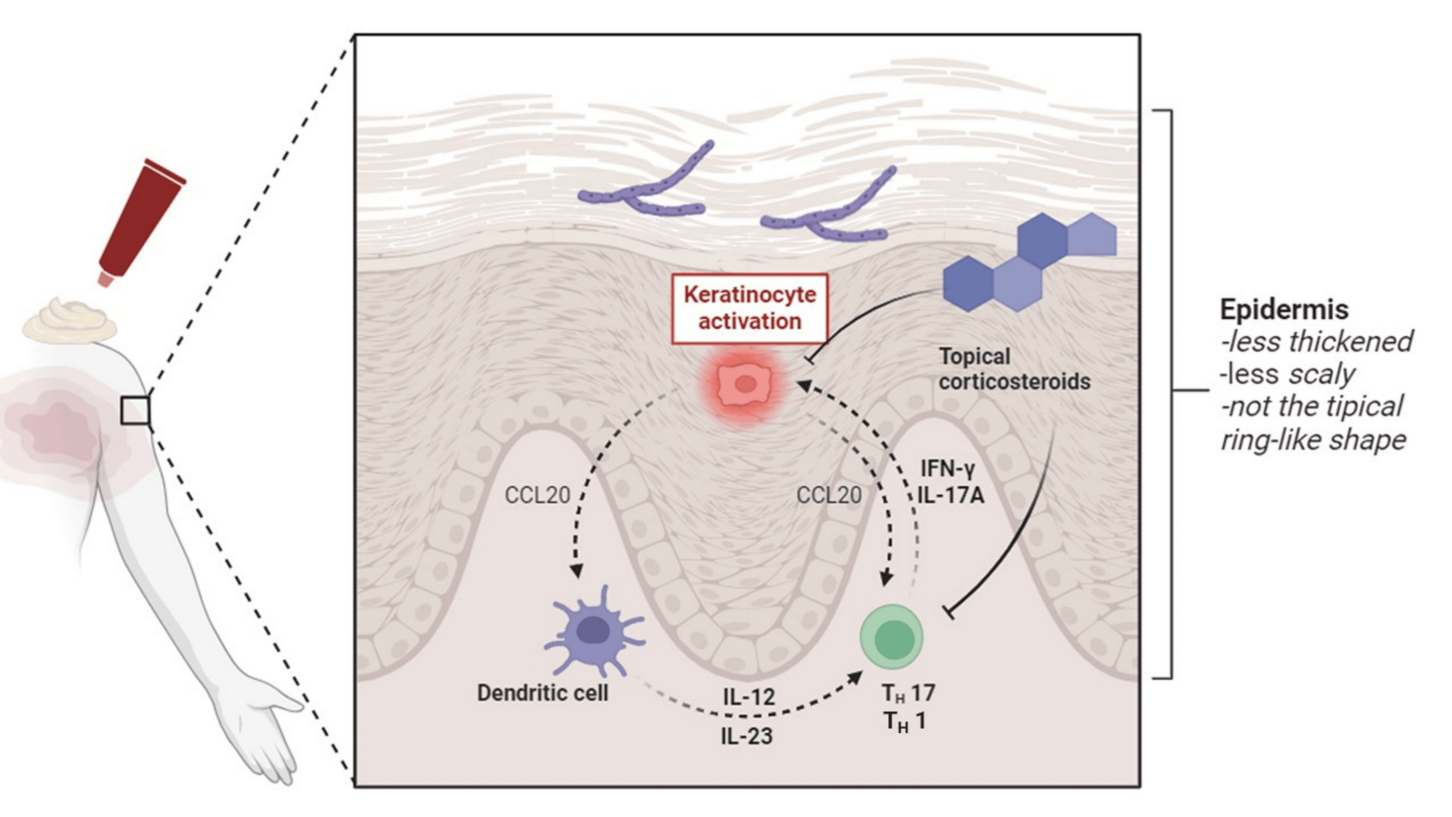
Impact of glucocorticoids on the immune response and pathogenesis of tinea incognito This figure illustrates the proposed mechanism by which long-term glucocorticoid use impairs antifungal immunity in tinea incognito (TI). Glucocorticoids suppress the Th17 and Th1 immune responses, which are crucial for clearing dermatophyte infections. The suppression of key cytokines like IL-17 and IFN-γ leads to weakened antifungal defenses and prolonged infection. Image credit: Jesús Iván Martínez-Ortega; created with biorender.com.

The pathogenesis of TI and the impact of glucocorticoid use on dermatophyte infections remain poorly understood. Phan et al. demonstrated in mice that the absence of local glucocorticoid production in the skin leads to a psoriasiform inflammatory response, driven by an activated Th17/Th1 axis, with increased involvement of unconventional T cells producing IFN-γ and IL-17A [17]. Interestingly, Heinen et al. showed that in dermatophyte infections, when both IL-17 and IFN-γ pathways are deficient, mice experience persistent superficial infection, which aligns with the clinical progression of tinea incognita under chronic glucocorticoid use, where superficial fungal spread is common [18]. Since Th17 and Th1 responses are essential for effective antifungal immunity and clearance [19], we hypothesize that long-term glucocorticoid use may impair these immune pathways. By suppressing key cytokines such as IL-17 and IFN-γ, glucocorticoids could weaken the body's antifungal defenses, leading to increased susceptibility and prolonged fungal infections. Investigating the effects of glucocorticoids on these immune mechanisms could reveal novel therapeutic strategies to balance their use in antifungal defense while minimizing adverse effects (Figure 3).

We also believe that the findings of Phan et al. may provide insight into the clinical phenomenon of tachyphylaxis, which refers to the gradual loss of therapeutic efficacy over time [17]. This effect is often seen when patients increase the frequency and dosage of steroid application in an attempt to achieve the same relief as the pruritic relief they initially experienced. The negative feedback from external steroids may suppress the skin's endogenous production of glucocorticoids, ultimately lowering the basal levels of local steroid effects after prolonged use [17].

Since physicians and family doctors are often the first to see these patients, it is crucial to consider the possibility of underlying chronic conditions, such as diabetes or insulin resistance, particularly if these conditions have not yet been diagnosed. TI should be viewed as a potential indicator of such underlying issues, as it can sometimes be the initial sign of a chronic condition that remains unidentified, as illustrated by this case [20].

The primary treatment for TI involves discontinuing corticosteroids or other immunosuppressive therapies. Topical antifungals are the primary treatment for mild, localized tinea infections. Terbinafine, clotrimazole, miconazole, or econazole creams or gels are applied twice daily for several weeks until the infection resolves. For more severe or extensive cases, or when hair-bearing areas are involved, oral antifungals like terbinafine, itraconazole, or fluconazole are more effective than griseofulvin. Treatment typically lasts a few weeks to several months, depending on severity [3,5,21].

Associated conditions, whether pre-existing or newly diagnosed, should be addressed holistically. For instance, obesity should be managed through nutritional counseling, and when necessary, with pharmacological treatment or surgery. Measures to reduce humidity and wearing appropriate clothing are essential. Additionally, conditions like diabetes or insulin resistance require appropriate medical management to optimize overall treatment outcomes [12,20].

This case raises the question of whether insulin resistance, like diabetes, can predispose individuals to dermatophyte infections. We could not find studies that specifically differentiate between insulin resistance and diabetes in this context, as most patients with diabetes also exhibit insulin resistance, but not all patients with insulin resistance develop diabetes. It would be valuable for future research to explore this potential link in more detail. A key limitation of this article is that while the HOMA-IR index is commonly used for the initial screening of insulin resistance, it would be advisable to confirm the diagnosis with additional tests. 

Furthermore, while we focus on pathogenic factors such as tachyphylaxis, which can lead to continued corticosteroid use and clinical misdiagnosis and mistreatment, we do not address cultural and governmental policy factors. The over-the-counter availability and low cost of corticosteroids significantly contribute to the prevalence of misdiagnosis and inappropriate treatment. Although these factors are not the primary focus of this article, they are important considerations in understanding the broader context of dermatophyte infections.

## Conclusions

Tinea incognito (TI) continues to present a significant diagnostic challenge due to its atypical manifestations, often exacerbated by the inappropriate use of corticosteroids or immunosuppressive therapies. This case emphasizes the critical need for clinicians to recognize altered fungal infections, especially when certain mistreatments worsen the condition. A comprehensive diagnosis relies on an integrated approach, including thorough patient history, mycological testing, and meticulous clinical examination. Chronic glucocorticoid use compromises the immune response by diminishing key cytokines like IL-17 and IFN-γ, facilitating persistent fungal spread. Furthermore, the occurrence of TI should raise suspicion for underlying conditions such as obesity, insulin resistance, or diabetes, even when the patient is unaware of these issues. Timely identification and appropriate antifungal treatment are paramount in preventing complications and improving patient outcomes.
